# Effect of ozone stress on the intracellular metabolites from *Cobetia marina*

**DOI:** 10.1007/s00216-020-02810-6

**Published:** 2020-07-16

**Authors:** Junjie Li, Christoph Rumancev, Holger V. Lutze, Torsten C. Schmidt, Axel Rosenhahn, Oliver J. Schmitz

**Affiliations:** 1 Applied Analytical Chemistry & Teaching and Research Center for Separation, University of Duisburg-Essen, Universitaetsstr. 5, 45141 Essen, Germany; 2 Analytical Chemistry – Biointerfaces, Ruhr University Bochum, Universitaetsstr. 150, 44780 Bochum, Germany; 3 Instrumental Analytical Chemistry and Centre for Environmental and Water Research (ZWU), University of Duisburg-Essen, Universitaetsstr. 5, 45141 Essen, Germany; 4 Technical University of Darmstadt, Department of Civil and Environmental Engineering, Institut IWAR, Franziska Braun Str. 7, 64287 Darmstadt, Germany; 5 IWW Water Centre, Moritzstr. 26, 45476 Mülheim an der Ruhr, Germany

**Keywords:** *Cobetia marina*, Oxidative stress, GCxGC, Bacterial metabolome

## Abstract

A GCxGC-MS system was employed with a non-polar × mid-polar column set for the metabolic non-target analysis of *Cobetia marina*, the model bacteria for marine biofouling. *C*. *marina* was treated with ozone to investigate the intracellular metabolic state change under oxidative stress. A minimal inhibitory concentration test was involved to guarantee that the applied ozone dosages were not lethal for the cells. In this study, non-target analyses were performed to identify the metabolites according to the NIST database. As a result, over 170 signals were detected under normal living conditions including 35 potential metabolites. By the comparison of ozone-treated and non-treated samples, five compounds were selected to describe observed trends of signals in the contour plots. Oleic acid exhibited a slight growth by increasing ozone dosage. In contrast, other metabolites such as the amino acid l-proline showed less abundance after ozone treatment, which was more evident once ozone dosage was raised. Thus, this work could provide a hint for searching for up/downregulating factors in such environmental stress conditions for *C*. *marina*.

**Electronic supplementary material:**

The online version of this article (10.1007/s00216-020-02810-6) contains supplementary material, which is available to authorized users.

**Figure:**
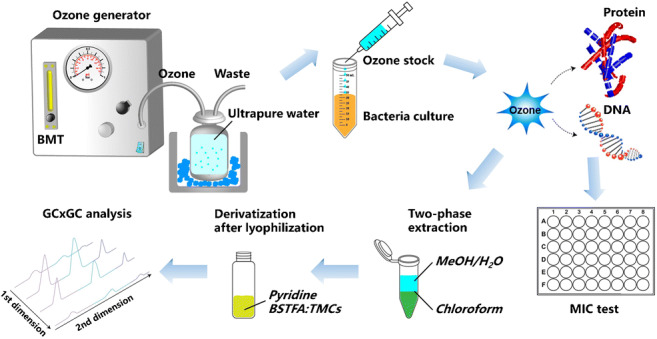
Graphical abstract

## Introduction

In general, microorganisms could optimally survive and reproduce due to the adaption to the normal environments [1]. However, the balance in such optimum condition could be broken by any extreme change, which was considered a kind of stress and might lead to lag time increase, growth rate reduction, and even cell death [2, 3]. Those stresses might include cold or heat shock [4, 5], hyperosmotic pressure [6], acid or organic solvent stress [7, 8], and oxidative stress [9, 10]. Compared with others, oxidative stress works non-physically but leads to oxidative damage via the accumulation of reactive oxygen species (ROS), which influences the lipids, nucleic acids, and proteins and then causes cell toxicity [11]. Typical ROS include superoxide anion (O_2_^−^), hydrogen peroxide (H_2_O_2_), and hydroxyl radicals (OH^•^) [12], which could disturb the redox reaction balance in different biological targets [11, 12].

**Fig. 1:**
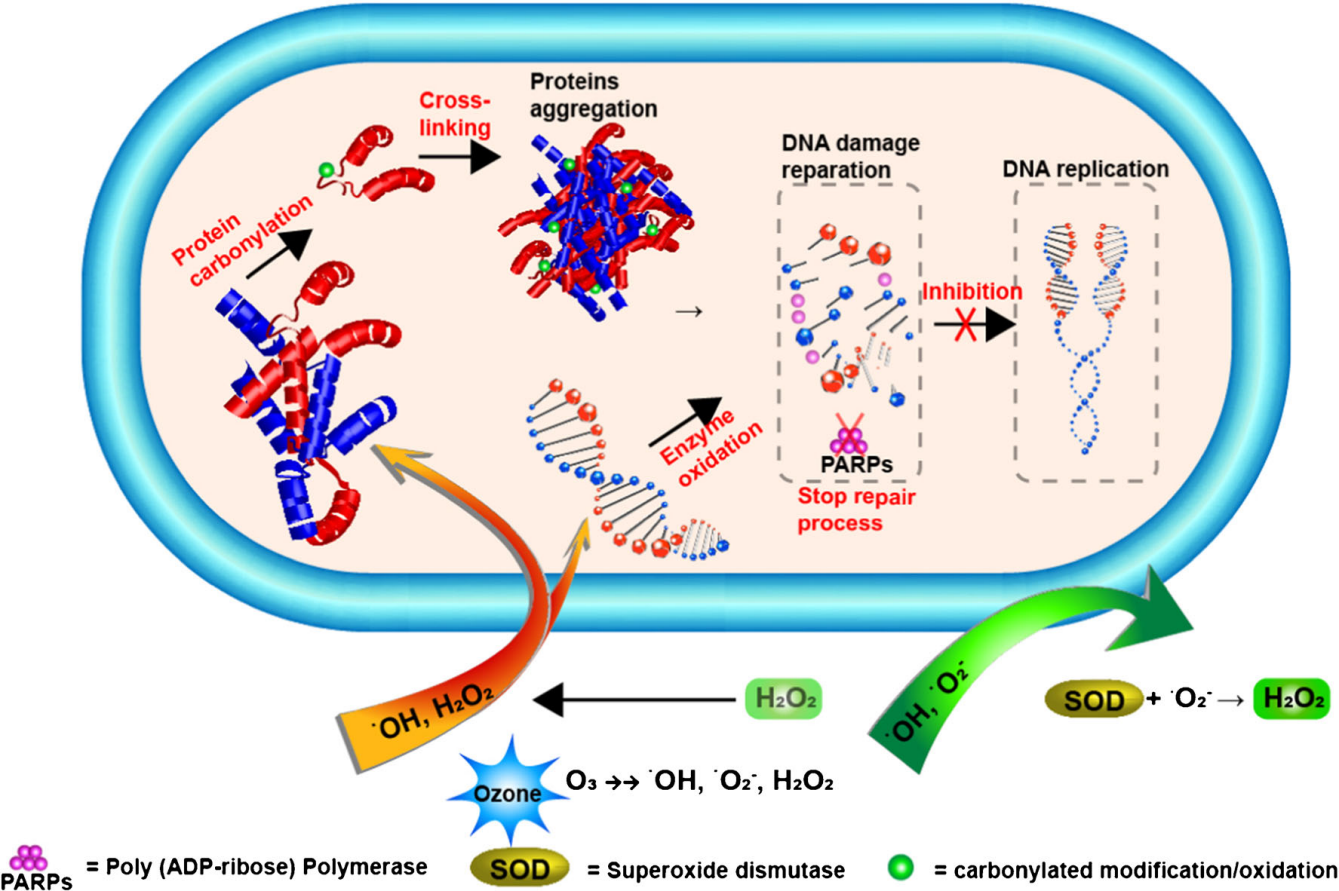
Decomposition of ozone in aqueous situation. Bacterial cell membrane, intracellular proteins, and DNA are the biological oxidized targets for the decomposed radicals

Ozone, as a powerful oxidant, has been widely used and considered to be one of the most effective antimicrobial agents since the last decades [13, 14]. Once added into aqueous solutions, ozone was decomposed rapidly, which generated superoxide radicals (^•^O_2_^−^), hydroperoxy radicals (HO_2_^•^), and OH^•^ radicals [15]. As shown in Fig. 1, these radicals would attack the bacterial cell surface by oxidizing mainly two groups—polyunsaturated fatty acids and amino acids from peptides, enzymes, or proteins.

Once passing through the membrane, it also leads to DNA or intracellular protein damages, which impacts the reparation and transcription and then might result in cell lysis or death [16, 17].

*Cobetia marina*, a Gram-negative marine bacterium, was first proposed in 1971 by Cobet et al. [18]. Considerable research over the past few decades has indicated its feasible features as a biofouling model system in marine or similar circumstance [19]. However, previous studies, especially in the last decade, barely focused on metabolite aspect—the unique chemical fingerprints regarding certain cellular processes [20], which might help to understand its metabolic changes according to the antibiofouling treatment or artificial environmental stress.

In recent years, metabolome, as the downstream of genome, has attracted increased attention, which describes and reflects the cellular activities more vividly and specifically at a functional level [21, 22]. Those researches for the metabolites with low molecular weight were contributed to understand the physiological state under certain circumstance or environmental stress [23–27]. On the other hand, with the development of analytical techniques such as multi-dimensional chromatography coupled to mass spectrometry (MS), the microbial fingerprinting qualitative analysis could be achieved with high coverage due to the great improvement of selectivity and separation power [28–30]. Therefore, the utilization of comprehensive 2D gas chromatography (GCxGC) offers a better chance for the untargeted analysis to understand the metabolic pathways or discover those potential biomarkers [31, 32].

To our knowledge, there is not much work on the study of the antibiofouling treatment of *C*. *marina* in relation to its metabolites. Tweeddale et al. evaluated the response in *Escherichia coli* to stresses induced by ROS, which proposed the metabolite variation in valine and glutathione levels [33]. However, this excellent work shows one limitation of the used TLC system, because it was hard to get a clear overview of the intracellular state before and after the stress.

In this study, a GCxGC-MS system was used for the metabolic non-target analysis. *C*. *marina*, the model bacteria for biofouling, was cultured and investigated for the intracellular metabolic state change under the ozone stress. Such stress was created by spiking aqueous ozone stock solution produced by an ozone generator. Before analysis, a modified sample preparation procedure was applied to guarantee that the majority of intracellular metabolites were collected with less contaminants and with as little as possible changes of metabolome during sample preparation.

Furthermore, a minimal inhibitory concentration (MIC) test was performed with different dosages comparable with those spiked in the real sample. With the help of the software named “GasPedal,” the states before and after the ozone stress were carefully compared by the contours plots.

## Experiments

### Chemicals

Deionized water was purified using a Millipore system (Millipore, Milford, MA, USA). MS-grade methanol and derivatization reagents including pyridine, methoxyamine hydrochloride, and *N*,*O*-bis(trimethylsilyl)trifluoroacetamide (BSTFA) with 1% trimethylchlorosilane (BSTFA:TMCS, 99:1) were obtained from Sigma-Aldrich Chemie GmbH (Steinheim, Germany). Sodium chloride (NaCl) used for preparing cell washing and chloroform were purchased from Sigma.

For preparation of the ozone stock solution, ultrapure water was produced with Purelab Ultra system (ELGA LabWater, Celle, Germany). Ozone-containing gas was produced onsite with an ozone generator (BMT 802 X, BMT Messtechnik, Berlin, Germany; feed gas: O_2_ 6.0, Linde, Duesseldorf, Germany), which was bubbled into ice-cooled ultrapure water as shown in Fig. S1 in the Electronic Supplementary Material (ESM). 10 mM of indigotrisulfonate purchased from Sigma was dissolved in ultrapure water.

### Bacterial culture

The dried culture of *Cobetia marina*, strain DSM 4741, was obtained from DSMZ (“Deutsche Sammlung von Mikroorganismen und Zellkulturen” GmbH, Braunschweig, Germany), an aerobic, Gram-negative bacterium. The prepared stock solutions were stored frozen at − 70 °C, using marine broth (MB) (2216, Difco, Augsburg, Germany) with 20% glycerol. By adding 2% Bacto agar (Difco) to MB, marine agar (MA) plates were prepared for bacterial streaking, which then stored at 4 °C for 3 weeks. For each sample used for the experiments, a single colony from an agar plate was inoculated into 20 mL sterile MB and allowed to grow on a platform shaker (65 rpm) at room temperature. As shown in Fig. S2 (see ESM), the optical density (*k* = 600 nm) of *C*. *marina* reached the stationary phase with an optical density of OD_600_ = 1 after approximately 12 h (overnight) growing [34].

### Ozone treatment

The concentration of the ozone in solution was determined by UV absorption at 258 nm (ozone stock solution, *ε* = 2950 M^−1^ cm^−1^) using a UV-1650PC spectrophotometer (Shimadzu, Kyoto, Japan) and using the the indigo method carried out at pH 1.6 and ambient temperature (ozone depletion) [35]. The ozone depletion test was performed according to Bader et al. [36]. The ozone stock solution was spiked with a gastight syringe (Hamilton, Reno, USA) to obtain ozone concentration in the bacterial sample of 500 μM and 600 μM. The samples were then incubated for 10 min before further preparation. A bacterial sample without ozone dosage was used as a control sample. There are two parallels of each ozone dosage prepared for the validation (*N* = 2).

### Sample preparation

Unless otherwise described, all operations were performed at 0 °C and all used reagents and containers were set to 0 °C. After the ozone stress treatment, the bacterial sample was quenched immediately to stop the metabolic activity. At first, 100 mL deionized water containing 0.85% NaCl was frozen into small ice bulks instead of an ice block as done in Wang’s work [37], which provides more contact area for the bacteria to optimize the quenching effect. After this, the ice bulks were transferred into a 200-mL plastic cubic bottle. A 100-mL ozone-treated culture was poured into the bottle directly after incubation. Thereafter, the bottle was shaken several times to mix well and placed at − 80 °C for 3 min. Then, the quenched culture was transferred into 50-mL prechilled Falcon tubes in aliquots of 20 mL. To get rid of the MB, each aliquot was centrifuged at 2000*g* at 4 °C. The cell pellets were washed two times with 30 mL and 1.5 mL phosphate-buffered saline (PBS) solution followed by centrifugation at 8000*g* for 3 min to remove the PBS. Before extraction of intracellular metabolites, the disruption of the bacterial cells was performed by transferring washed cell pellets together with 0.25 mL chloroform and 0.25 mL MeOH/H_2_O (21:79, v/v) into a lysis tube. Then, the cells were disrupted by SpeedMill PLUS (Analytik Jena AG, Jena, Germany) at a continuous mode for 1 min. After disruption, the whole mixture was collected and transferred into a 1.5-mL centrifuging tube. After that, the metabolites were extracted by two-phase extraction with a mixture of 0.25 mL chloroform and 0.25 mL MeOH/H_2_O. It is worth to mention that the disruption reagents are the same as those used for extraction, which obviate the removal of disruption reagents and minimize the loss of metabolites. Subsequently, the extraction mixture was vortexed for 30 s and centrifuged at 13,360 *g* for 10 min. A total of 400 μL from the upper phase was collected, which followed by re-extracting the lower phase with 0.5 mL MeOH/H_2_O. Two 400-μL extracts were combined and stored at − 80 °C. To get rid of water, the frozen sample was then lyophilized at approximated − 45 °C in an Alpha 1–2 LD plus vacuum lyophilizer (Christ, Osterode, Germany). The dried samples were stored at − 80 °C before derivatization. The derivatization was performed in two steps. First, 100 μL pyridine (with 25 mg/mL methoxyamine hydrochloride) was added and incubated at 60 °C for 60 min. In a second step, 100 μL TMS reagent (BSTFA:TMCS, 99:1) was added and the mixture was heated at 60 °C for 60 min. In summary, the quenching process was necessary because it is environmentally friendly and less harmful to the bacterial cell than organic solvents. The washing step was applied to ensure that these extracellular disturbances and impurities were removed before the cell was broken down. To determine the metabolites by GCxGC, the extracted metabolites were partially derivatized prior to analysis.

### Instrumentation

The analysis was performed using a GCxGC-quadrupole-MS (Shimadzu QP-2010). In the first dimension, a Rxi-5sil MS column (30 m × 0.25 mm × 0.25 μm; Restek, USA) was used. A middle polar Rxi-17sil MS column (1 m × 0.15 mm × 0.15 μm; Restek, USA) was applied in the second dimension. The modulation time of the dual-jet cryogenic modulator was 3.8 s. Helium was used as the carrier gas with a constant flow of 1.09 mL min^−1^. A total of 1 μL of derivatized sample was injected at 280 °C with a split ratio of 1:1. The oven for both columns was heated up from 80 °C (5 min hold) to 300 °C (15 min hold) at 5 °C min^−1^. The transfer line and ion source were kept at 310 °C and 200 °C, respectively.

### Software

GC Image from Lincoln (Nebraska, USA) was applied for visualizing the raw data from GCxGC-MS, which generates 2D contour plots. GasPedal developed by DECODON (Greifswald, Germany) was used for the inter-contour plot comparison and calculation.

## Results and discussion

### Repeatability of sample preparation

The repeatability of the bacterial sample preparation method was investigated for different culture volume groups (5 mL, 10 mL, 30 mL) in triplicates with OD_600_ ≈ 1 in the stationary phase. To get an overview of the contour plots, the GasPedal software was employed for the comparison. The so-called fusing images combine the features including spot position and intensities from selected images. As shown in Fig. S3 (see ESM), the signals with relative standard deviation (RSD in %) less than 30% were 150 (94.9% of all signals) in the 5 mL culture group, 141 (94.6%) in the 15 mL culture group, and 159 (96.3%) in the 30 mL culture group. The standard deviation is comparable with the results from Maifiah et al. for their study on *Acinetobacter baumannii* with RSD% of 22% for metabolites extracted by chloroform/methanol/water [38].

### Identification of metabolites by GCxGC-MS

**Fig. 2:**
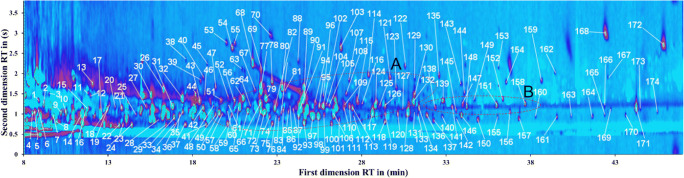
Contour plot from GC Image. Intracellular metabolites of *C*. *marina* were detected as spots in the plot. The *x*-axis represents the retention time (min) for the first dimension. The *y*-axis represents the retention time (s) for the second dimension

With the instrumental parameters mentioned above, 1 μL of derivatized sample was injected into the GCxGC-MS for the metabolic analysis in this study. Generally, at first, the raw data were visualized in GC Image as a contour plot, as shown in Fig. 2. More than 170 intensive signals could be detected, which implies an overview of intracellular metabolic state of *C*. *marina* as a “snapshot” before ozone treatment. The retention times in both dimensions were employed to search those intensive spots on the total ion current (TIC) chromatogram. The MS spectra were compared with NIST library, which offers suggested substances with match factors. After the comparison with literature and NIST database, those compounds as potential metabolites with match factors ≥ 80 were selected and are listed in Table 1.

**Table 1:** The potential intracellular metabolites of *C*. *marina* according to the NIST library

Spot number	Suggested metabolites	Retention times		Match factor
		1st D (min)	2nd D (s)	
4	Octanol	8.54	2.54	86
7	Propanoic acid	9.81	2.42	92
11	Pentanoic acid	11.90	2.98	91
14	Glycine	11.52	2.50	93
17	Butanoic acid	12.66	2.46	93
19	Isoleucine	13.17	2.74	80
25	Methylmalonic monoamide	14.75	2.46	80
31	Succinic acid	16.90	2.66	91
48	Glutaric acid	19.37	2.70	87
50	l-Cystine	19.69	2.54	80
57	Beta-alanine	19.88	2.50	82
59	l-Homoserine	20.45	2.46	80
61	Threonine	20.26	2.66	81
65	Aminomalonic acid	20.96	2.70	90
66	Malic acid	21.40	2.58	91
68	l-Proline	21.72	3.78	80
71	l-Methionine	22.16	2.74	89
72	l-Aspartic acid	22.22	2.58	87
78	Alanine	22.86	3.30	91
90	l-phenylalanine	24.76	2.90	86
91	Glutamic acid	24.57	2.62	83
106	l-Lysine	26.53	2.50	89
112	d-Galactose	27.73	2.46	80
117	l-Ornithine	28.87	2.50	93
120	d-Glucose	30.39	2.50	86
121	Adenine	29.89	3.38	84
132	l-Tyrosine	31.34	2.78	92
133	Octadecanamide	31.60	2.46	80
138	Eicosane	32.61	2.62	80
143	Oleanitrile	34.13	3.26	88
148	Docosane	34.51	2.66	89
149	Myristic acid amide	34.95	2.86	80
151	Tetracosane	36.28	2.70	90
152	Oleic acid	36.66	2.86	88
158	Hexacosane	37.99	2.74	87

The TMS group in those metabolites derivatized by trimethylsilylation was omitted in this table

Two zones, A and B, were circled in Fig. 2, which imply the different groups of metabolites. For example, as shown in Table 1, amino acids such as l-cystine (**50**) and l-aspartic acid (**72**) were located in zone A. In zone B, there was the alkane group including eicosane (**138**) and docosane (**148**). In addition, fatty acid metabolites such as butanoic acid (**17**), succinic acid (**31**), and oleic acid (**152**) were detected with high match factors. Similar to previous reported work [29], the homogenous distribution in different classes was validated for the intracellular metabolites of *C*. *marina*. Furthermore, nearly all the detected metabolites were separated well without co-elution. The orthogonality and optimized parameters thus reduce interference and bring advantages for the qualification and quantification of such derivatized samples.

### Minimal inhibitory concentration test

In this study, ozone was added for simulating environmental living stress. Therefore, the dosage of ozone was critical to create sufficient stress without killing or destroying the bacteria. To ensure this, a test according to Irith et al. was performed [39].

As stated in Table S1 (see ESM), different dosages of ozone were spiked from 170 to 1000 μM into different 48-well plates (each with 500-μL bacterial culture). The initial volume for the first well of each row was 500-μL bacterial culture plus 500 μL spiked 1.5 mM ozone stock solution. After mixing with pipette, 500 μL from the first well was transferred to the next well in the same row. That means in the second well, it would be 750-μL bacterial culture plus 250 μL spiked ozone solution. Similarly, 125 μL ozone solution was kept in the third well. This was performed until well number 6 (see ESM Fig. S4). At the end, the ozone stock solution in the wells was decreasing by mixing with increasing number of bacteria, which means less stress from ozone on the bacteria by transferring the diluted mixture. The 48-well plate was then shaken for 24 h at room temperature. As shown in Fig. S5 (see ESM), taking row A as an example, the colors of bacterial culture were increasingly turbid from A1 to A6. Less aggregation of bacteria was found from A1 to A5 compared with A6, which has the least ozone-stressed bacteria. It reveals that an increasing living activity could be found by decreasing stress. Influence of ozone stress treatment on metabolic activity might lead to the reproduction declining of bacteria even after a long period. For A1 and A8, a clear difference could be observed between with and without ozone dosages, which could be due to the suppressed growth of bacteria by the ozone treatment and to a reduced chance for the bacteria to aggregate together.

To confirm the bacterial activity and cell integrity after ozone treatment, the bacteria from different wells in the first row of each plate were streaked and re-cultivated on the MA plate. As shown in Fig. S6 (see ESM), it was obvious that all bacteria could regrow on the agar plate similar to the growth control sections. Therefore, it was proven that *C*. *marina* was stressed by ozone and not inactivated by cell membrane damage directly. Accordingly, the intracellular state might change to resist the stress for surviving during the attack from ozone.

### Intracellular state variation induced by ozone stress

**Fig. 3:**
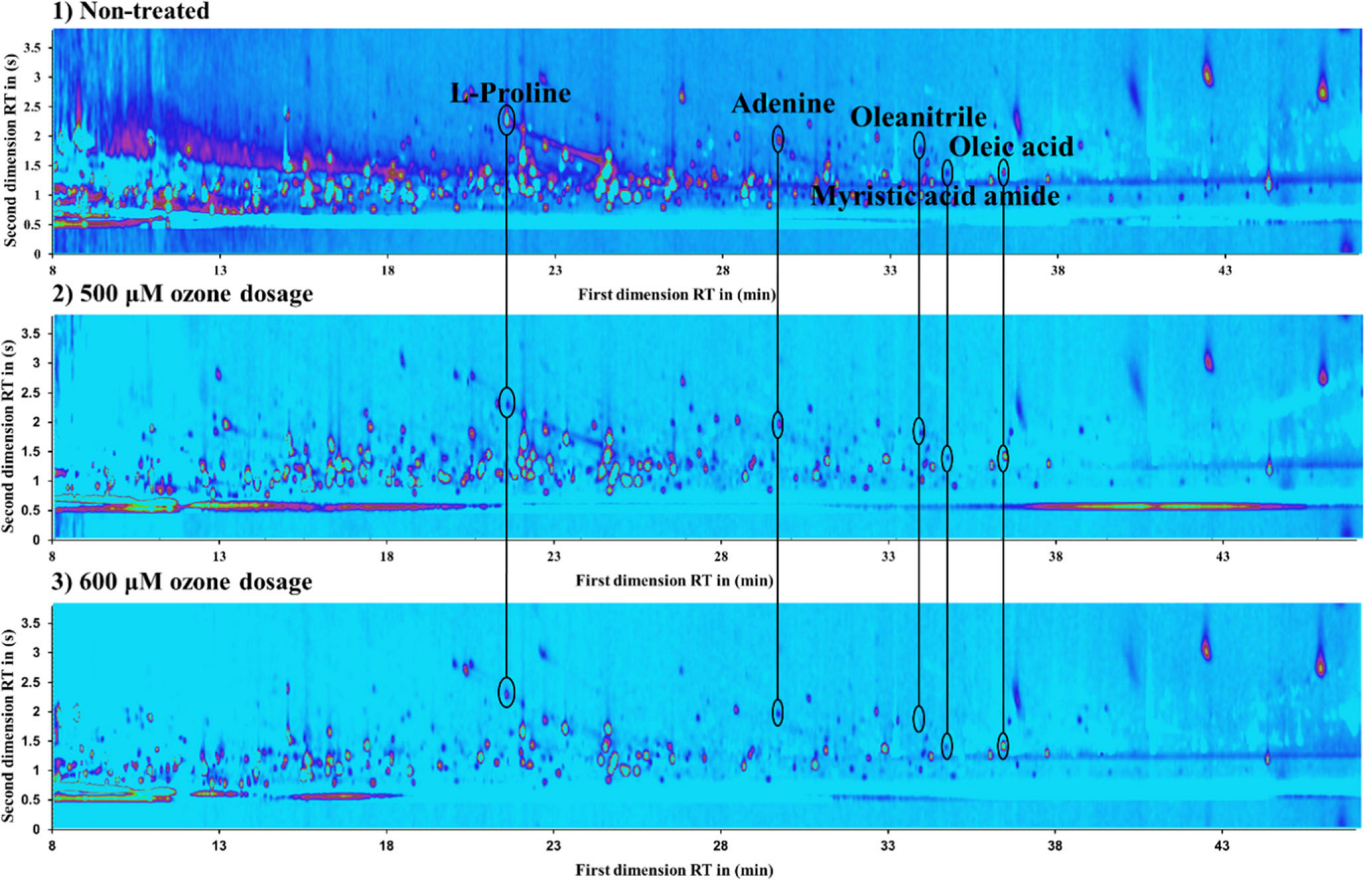
The contour plots with potential intracellular metabolites of *C*. *marina* non-treated (1) and treated with 500 μM (2) and with 600 μM (3) ozone

Based on the conclusion of the MIC test, the ozone treatment was not lethal but obviously influenced the living state of *C*. *marina*. To verify such physiological change, a non-target analysis with GCxGC-MS at a scan mode was performed. Three contour plots of ozone-treated (two different concentrations) and non-treated samples were selected for further comparison (Fig. 3). There is a clear trend that the detected signals became less and weaker once the ozone treatment was applied. Furthermore, the effect was amplified in some cases when the ozone dosage was increased from 500 to 600 μM.

In Fig. 3, five spots, presented in each contour plot, were circled and identified according to the mass spectra. The suggested substances are listed in Table 1, which were from different classes such as fatty acid, amino acid, nucleotide, sugar, and nitrile.

Hazel et al. reported that the fatty acid composition in the cell membrane could improve the ability to survive under the physical change of living condition [40]. Recently, it was also proved that the whole cell–derived fatty acid of some bacteria such as *E*. *coli* and *Pseudomonas aeruginosa* were changed in constituents when exposed to different stresses and outer contaminants [41]. Similarly, compounds l-proline (**68**) and adenine (**121**) in Fig. 3 showed a contrary trend. For myristic acid amide (**149**), a reducing abundance was observed after oxidative stress by ozone. In contrast, oleic acid (**152**) showed an inverse activity in ozone-treated samples. The concentration of oleic acid, as a typical long chain fatty acid (LCFA), was found to grow slightly according to Belenky et al. [42]. They proposed that the cell toxicity of treating *E*. *coli* with three different antibiotics and its behavior were similar to those under the oxidative stress. Therefore, the oleic acid might be an upregulated metabolic factor under such ozone stress.

For the amino acid in Fig. 3, l-proline (**68**), a decrease in intensity was observed after ozone treatment. As described by Mudd et al. [43], the amino acid class could be easily attacked and oxidized by ozone. Moreover, the intensity of oleanitrile (**143**), a fatty nitrile derived from oleic acid, also decreased in the treated samples. In addition, the other four compounds, with the exception of oleic acid, showed a difference between 500- and 600-μM ozone dosage.

**Fig. 4:**
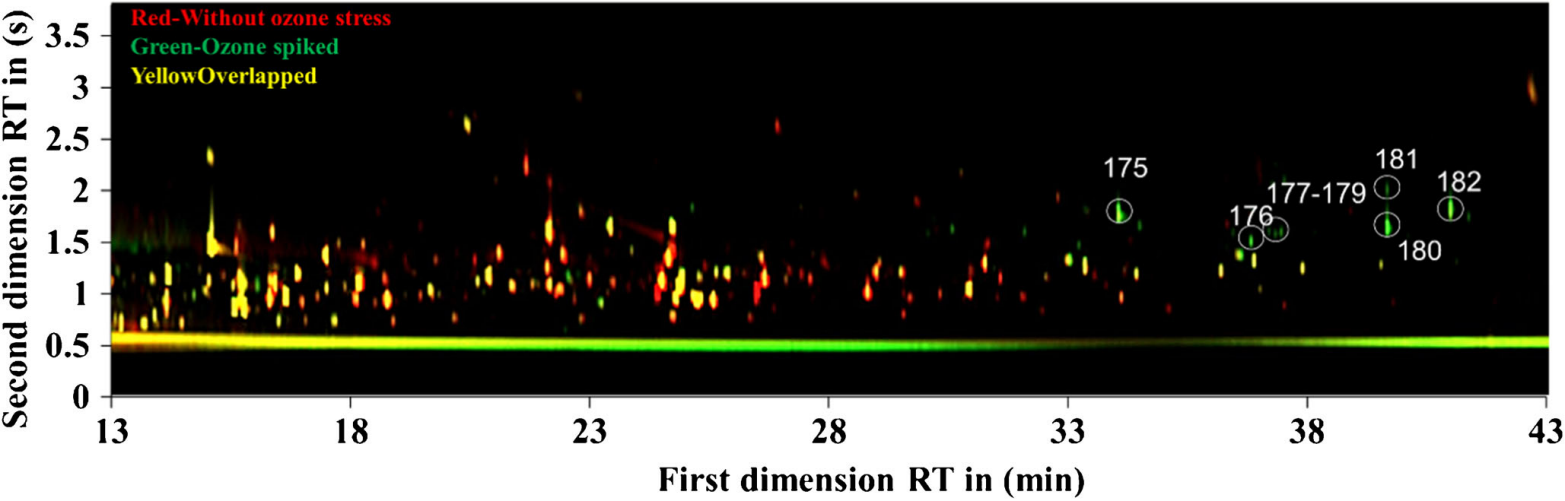
Comparison by overlaying the contour plots performed by the GasPedal software. Red: sample without ozone stress; green: sample with 600-μM ozone dosage; yellow: formed by the overlap of both contour plots

To get a more distinct overview of the difference between treated and non-treated samples, a warping image (Fig. 4) was generated by the GasPedal software to compare the contour plots of both samples.

Once certain spot occurred in both plots at the same position, the color of the spot turned into yellow at the overlap area. At first, more than half of the spots were overlapped (yellow) in Fig. 4 and were still present after the ozone treatment. However, some spots were yellow but surrounded by red color. This means that the abundance of these compounds was decreased. The other spots in red or green represent metabolites totally decreased or newly generated, respectively, due to the ozone treatment. As a result, the ozone effect was better visualized by such warp image, which allows the convenience for the fast inter-plot comparison without time-consuming observation with the naked eyes.

As shown in Fig. 4, the green spots represent those substances that have occurred due to ozone stress. According to the database, the suggested compounds are listed in Table S2 (see ESM). Such fatty aldehydes as cis-9-hexdecenal (**175**) and 9-octadecenal (**182**) could be intermediate metabolic products, which generated by the reduction of fatty acyl-CoA with acyl-CoA reductase. On the other hand, such aldehydes could not be reduced to fatty alcohols once lack of fatty aldehyde reductase. Yao et al. have proposed the production of fatty alcohols under environment stress [44]. However, the ozone stress might block the aldehyde reduction pathway, which remains the fatty aldehydes as the “snap” of fatty alcohol biosynthesis pathway. 13-Docosenamide was reported to be released by the bacteria in response to fluorescein quenching by Tamilmani et al. [45]. In this case, 13-docosenamide (**181**) was detected after the ozone treatment, which might be a stress response to the environment change. The other compounds in Table S2 (see ESM) do not show sufficient correspondence with the NIST database so that identification is not possible. The spectra of these unknown compounds are shown in Fig. S7 (see ESM).

## Conclusion

In this study, a method for bacterial metabolome analysis was successfully developed, including the bacterial sample preparation, ozone stress treatment, and 2D gas chromatography detection. The effect of ozone stress could be visualized and evaluated by the metabolic change of microbial state, which revealed by the intracellular metabolites. Several metabolites from *C*. *marina*, such as fatty acids and amino acids, were selected, which showed, with the exception of oleic acid, a decreasing trend by increasing ozone dosage. With the help of GasPedal, compounds such as 9-hexdecenal, 9-octadecenal, and 13-docosenamide were found in response of environment changes, which represents the ozone treatment in this study. Additional experiments for other antimicrobial stresses would be necessary for the confirmation of key up/downregulating metabolites.

## Electronic supplementary material

ESM 1 (DOCX 3614 kb)
