# Supplementary material for: Effect of ozone stress on the intracellular metabolites from Cobetia marina
Source: Anal Bioanal Chem. 2020 Jul 16;412(23):5853–61. doi: 10.1007/s00216-020-02810-6 (PMC7413921; doi:10.1007/s00216-020-02810-6)
Supplement: Supplementary file 1 — (DOCX 3614 kb) [file 216_2020_2810_MOESM1_ESM.docx]

**Analytical and Bioanalytical Chemistry**

**Electronic Supplementary Material**

**Effect of ozone stress on the intracellular metabolites from
*Cobetia marina***

Junjie Li, Christoph Rumancev, Holger V. Lutze, Torsten C. Schmidt,
Axel Rosenhahn, Oliver J. Schmitz


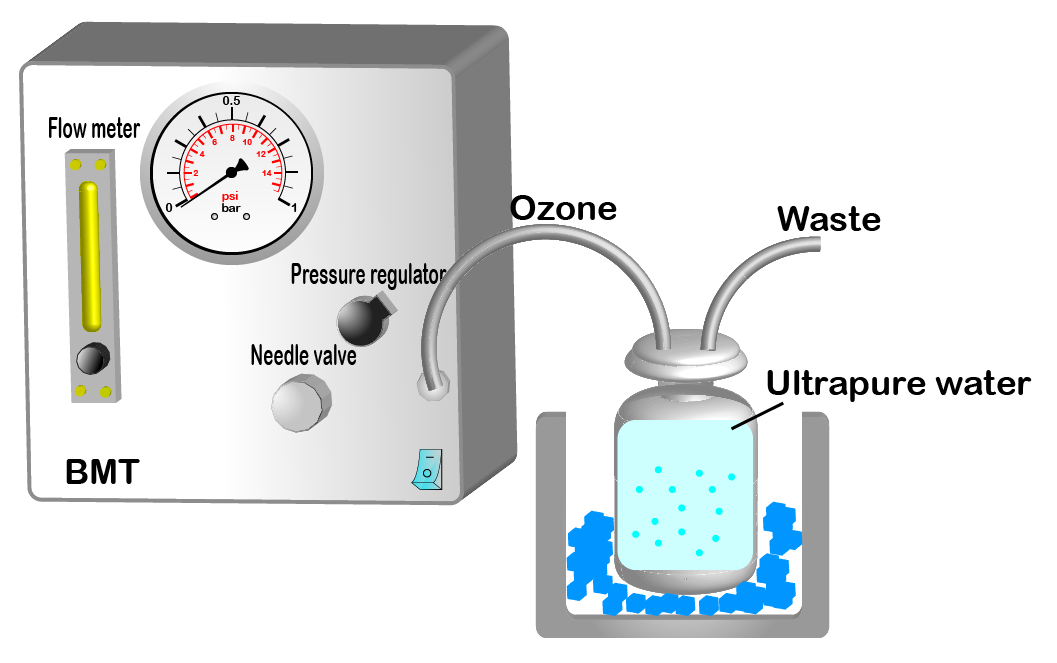


**Fig. S1** Ozone generator with ozone stock solution cooled by ice


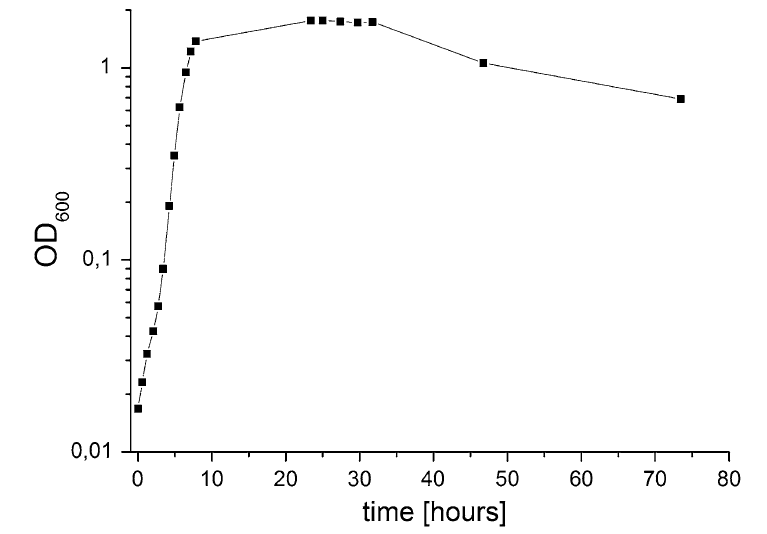


**Fig. S2** Growth curve of *Cobetia marina* in MB with optical density OD_600_ (measured at 600 nm wavelength)

**
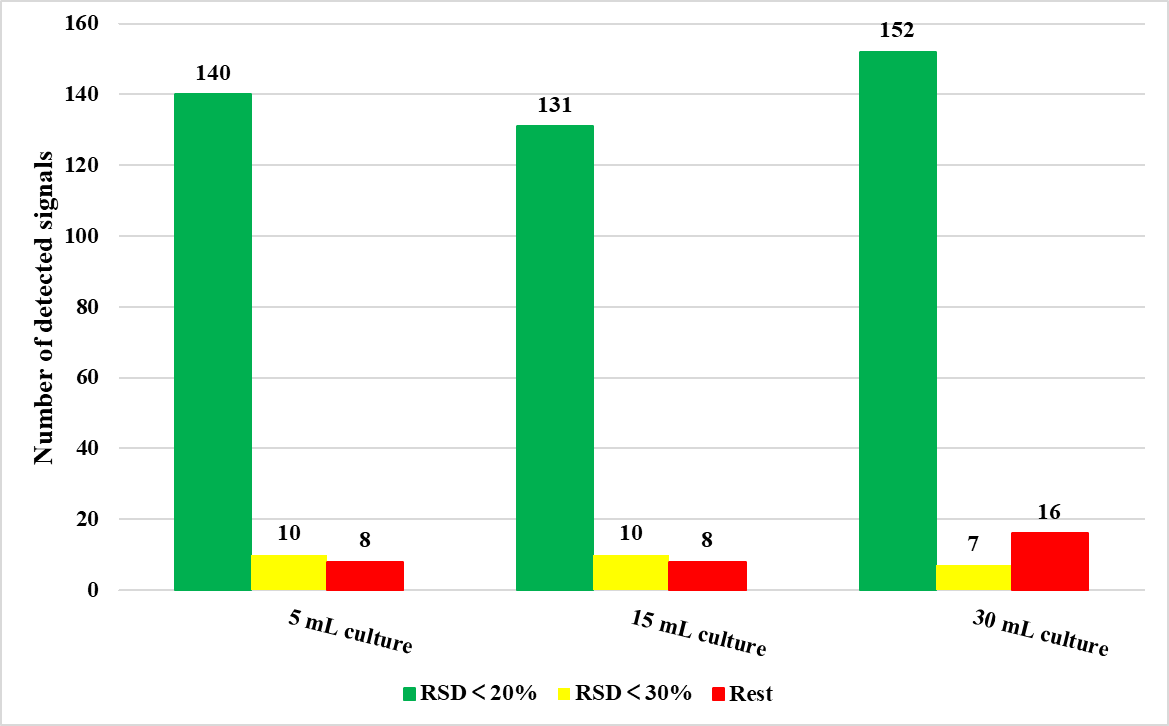
Fig. S3** Repeatability of three parallel bacteria sample in triplicates measurements. Relative standard of single signal in each parallel was indicated above the column bar


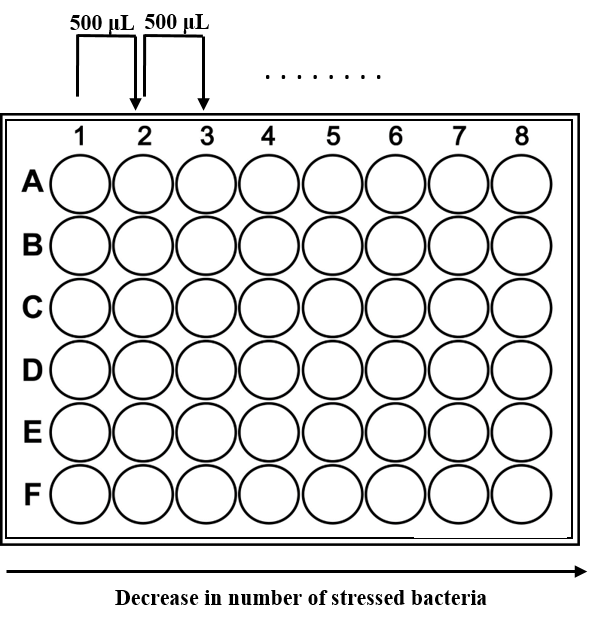


**Fig. S4** Workflow of MIC test. The wells in the first column was spiked with ozone stock solution. The initial volume of the bacteria culture in the each well was 500 μL. After spiking with 500 μL ozone stock and mixing, 500 μL of mixture were transferred to the next well in the same row, which was repeated from column 1 until column 6. From row A to F were six parallel repeats for the same initial dosage


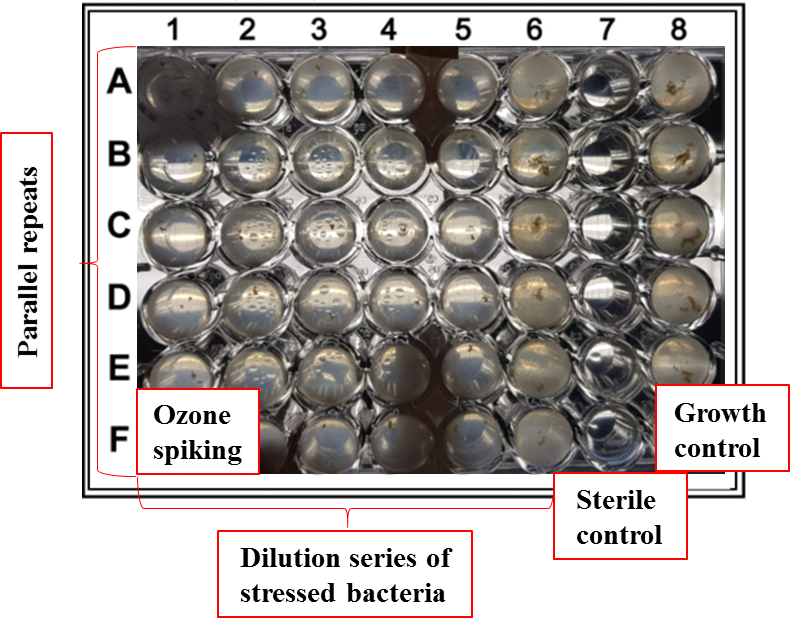


**Fig. S5** The 48-well plate with ozone dosage of 500 μM after 24h shaking. Column 1 to 6 were the dilution series by the ratio of 1:2. Column 7 was the sterile control without bacteria. Growth control at column 8 was bacteria culture without ozone treatment


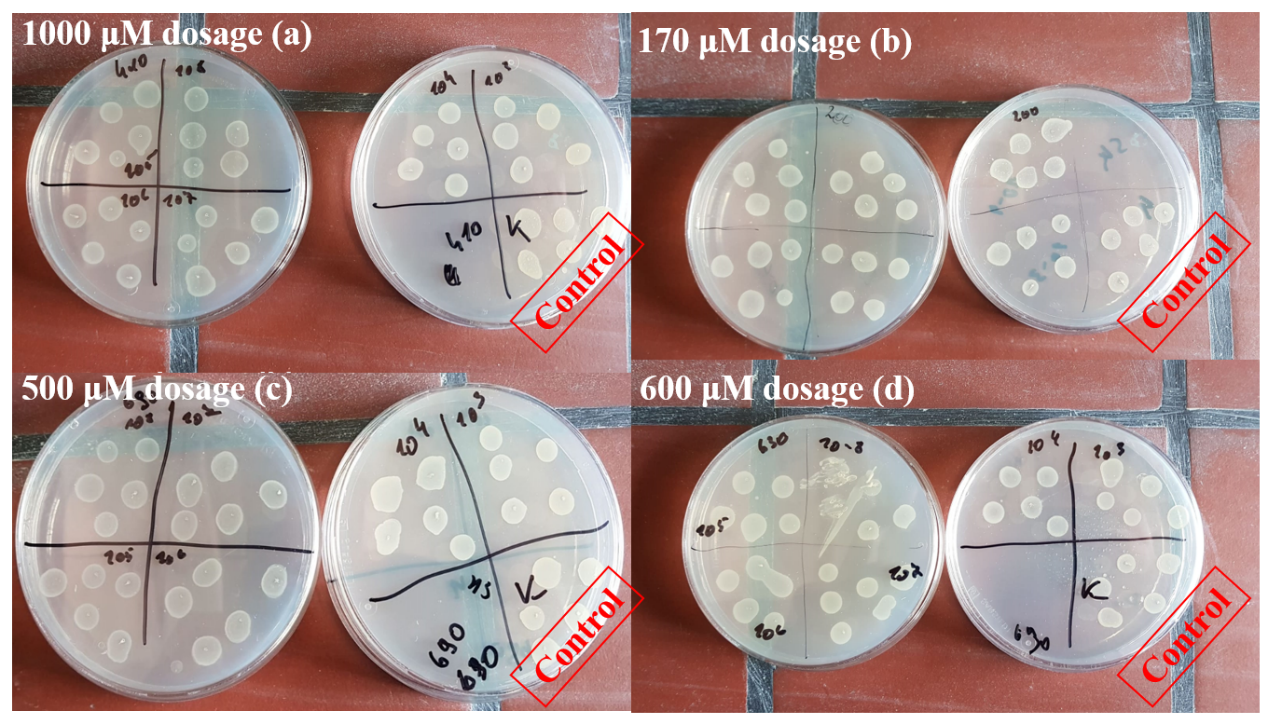


**Fig. S6** The re-growth of *C. Marina* culture on the MA agar plate after ozone treatment. Bacteria with ozone dosages 1000 μM (a), 170 μM (b), 500 μM (c) and 600 μM (d) were streaked in four different plates. The yellow-white dots were colonies formed by room-temperature incubation. Each plate contained four sections. On the first plate of each dosage, there were four parallels. On the second plate, there were another two parallels, growth control (in red) and sterile control (no colonies grown)


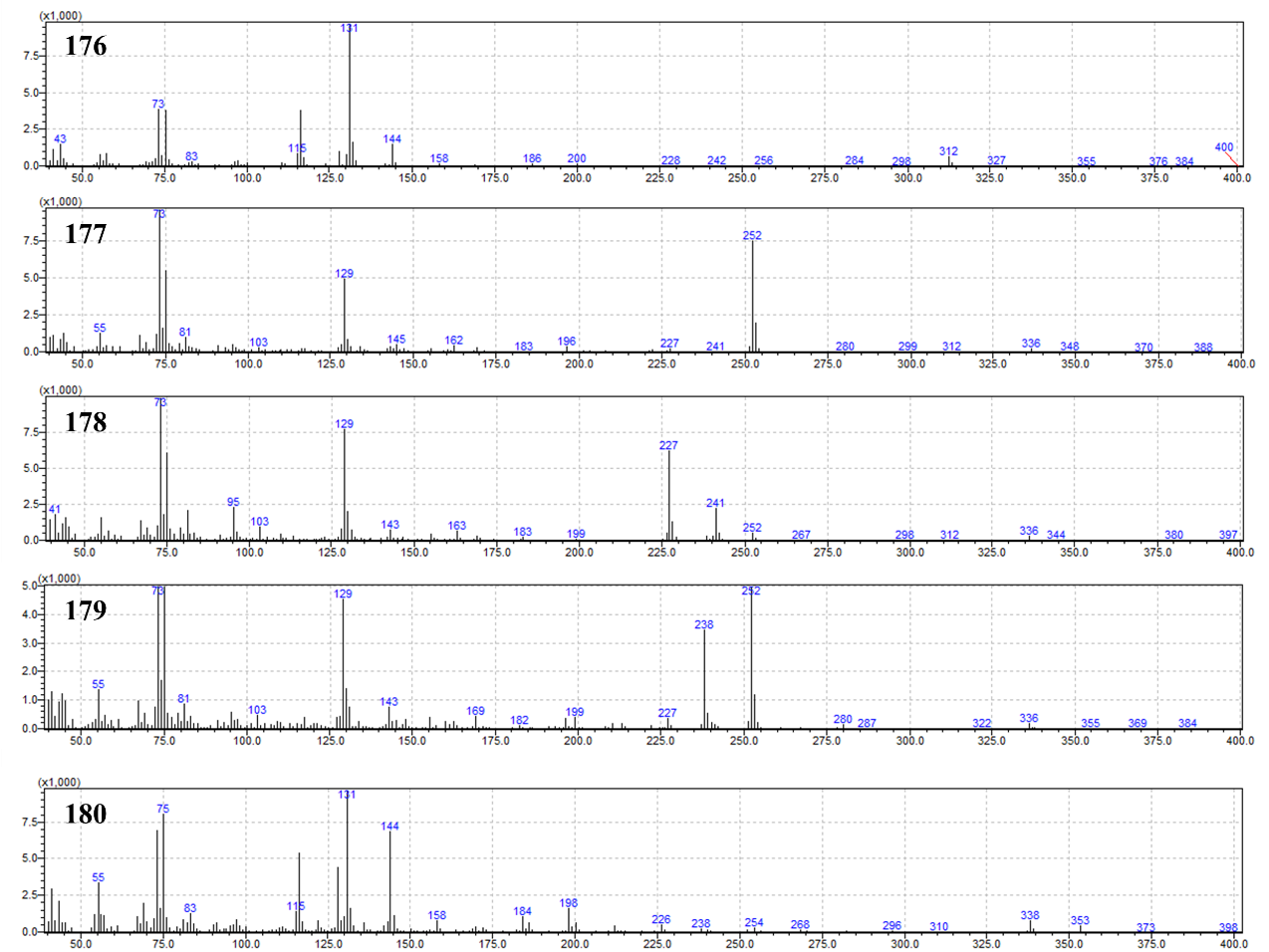


**Fig. S7** The spectra of unknown substances occurred after ozone treatment found with the help of GasPedal

| 48-well plate No. | Spiked Dosage  (concentration of O_3_ in bacteria culture) |
| --- | --- |
| 1 | 1000 μM |
| 2 | 170 μM |
| 3 | 500 μM |
| 4 | 600 μM |

**Table S1** The dosages of ozone in different 48-well plate for the MIC test

| No. | Substance | No. | Substance |
| --- | --- | --- | --- |
| 175 | cis-9-Hexdecenal | **176** | Unknown |
| 177 | Unknown | **178** | Unknown |
| 179 | Unknown | **180** | Unknown |
| 181 | 13-Docosenamide | **182** | 9-octadecenal |

**Table S2** The list of suggested substance according to the NIST database occurred after ozone stress
